# CDC Healthy Brain Initiative Action Institutes: Innovative Planning and Lessons Learned

**DOI:** 10.1093/geroni/igaa057.2544

**Published:** 2020-12-16

**Authors:** Heidi Holt

**Affiliations:** Centers for Disease Control and Prevention (CDC), Atlanta, Georgia, United States

## Abstract

This presentation will demonstrate an innovative strategic planning effort, coined Action Institutes (AI’s), which are designed to promote the implementation of CDC’s “The Healthy Brain Initiative’s State and Local Public Health Partnerships to Address Dementia” and the “Road Map for Indian Country.” Both of these documents outline how the champions of public health and their partners can create a statewide effort to promote brain health, increase early diagnosis, address cognitive impairment for individuals living in the community, and help meet the needs of care partners. The purpose of these 1-2-day AI’s is to familiarize leaders with the topic, encourage their adoption into current priority setting, and guide participants in creating action plans. The CDC’s Alzheimer’s Disease and Healthy Aging Program is conducting a series of these AIs, which are made possible through partnerships with the Association of State and Territorial Health Officials and the National Indian Health Board.

